# Disturbance function for soil disturbed state strength based on X-ray computed tomography triaxial test

**DOI:** 10.1371/journal.pone.0215961

**Published:** 2019-05-02

**Authors:** X. Li, S. J. Wang, B. H. He, Y. Frank Chen

**Affiliations:** 1 School of Resources and Environments, Southwest University, Chongqing, China; 2 School of Engineering and Technology, Southwest University, Chongqing, China; 3 Department of Civil Engineering, The Pennsylvania State University, Middletown, PA, United States of America; University of Science and Technology Beijing, CHINA

## Abstract

Soil is a porous, multiphase, and loose medium, which is prone to serious disturbance by the activity of biological livings, accompanied with cracks and macro pores. These phenomena greatly affect the strength of soil and the degradation of land. Two different quantifiable values from X-ray computed tomography (CT) images are usually used to define the disturbance function for soil disturbed state strength: mean density (*MD* value) and standard deviation (*SD* value). Two types of disturbance functions are investigated quantitatively by CT-triaxial testing using four samples with different macro-pore sizes (disturbed samples) and one sample without a macro-pore (undisturbed sample). It is found that the shear strength of three disturbed samples with the same macro-pore size is close to each other. As with the shear strength, the *SD* value and its defined disturbance function have a similar correlation with the cross-sectional area of macro-pore. However, the *MD* value and its defined disturbance function have no regularity with the macro-pore size. Therefore, the disturbance function expressed in terms of the *SD* value is deemed more reasonable and appropriate in analyzing the soil disturbed state strength using the quantitative CT morphometry. While, the disturbance function defined by the *MD* value has often been used to characterize a rock damage.

## Introduction

Soil is a porous, multiphase, and loose medium, which is prone to be seriously disturbed due to the growth of plant roots and the activity of biological livings, accompanied with cracks and macro pores being the two important structural characteristics of soil. The potential disturbance caused by the fracture and pore greatly affects the strength and stability of soil. However, the detailed geometry and variation of the disturbances cannot be observed in the traditional triaxial test because most of the disturbances occur inside the soil. X-ray computed tomography (CT) being a non-invasive and non-destructive technique was initially used in the medical field. CT images are constructed by the spatial distribution of the so called *CT value*, which is defined as follows:

$$CT\;value=1000\times \frac{\mu_{i}‑\mu_{w}}{\mu_{w}}$$ (1)

where *μ*_*i*_ is the coefficient of absorption at the scanning point, *μ*_*w*_ is the coefficient of absorption for water. Thus, the CT value of air should be -1000 because the coefficient of absorption for air is zero. Likewise, the CT-value for water is 0. CT images are presented with a shaded gray for low CT-value and light gray for high CT-value through the black to white range. It is well known that this CT-value, equal to the *MD*, is linearly related to material density.

The CT technique was first used in soil science by Petrovic et al [1]. to analyze the soil bulk density. In the recent decade, CT had been widely used to evaluate the evolution of the internal structure of soil by several researchers including Oda et al. [2], Pires et al. [3], Alshibli et al. [4], Schäffer et al. [5,6], Hall et al. [7], Willson et al. [8], Bruchon et al. [9], Naveed et al. [10] and Keyes et al. [11].

The X-ray computed tomography provides an insight into the soil structure to evaluate the evolution of pores or fractures using the image analysis. There are two quantifiable values from CT images: mean or *MD* value and standard deviation or *SD* value. The *MD* value represents the relative average density of the scanned sample section and the *SD* value represents the inhomogeneity of the density for the same section.

One kind of disturbance functions for soil disturbed state strength, *D*_1_, may be defined using the *MD* values as

$$D_{1}=\left|\frac{M_{0}‑M}{M_{0}+1000}\right|$$ (2)

where *M*_0_ is the *MD* valu*e* of soil sample at relative intact (RI) state and *M* is the *MD* value of soil at a certain disturbance. The *MD* values of air and water are -1000 and 0, respectively. Therefore, *D*_1_ = 0 when the sample is at RI state and = 1 when the sample is at fully disturbed (FD) state. Note that the FD state is a hypothetical state with the *MD* value set to be -1000 as the scanned section is fully filled with air.

Another disturbance function, *D*_2_, may be defined by the *SD* values as

$$D_{2}=\left|\frac{SD_{0}-SD}{SD_{0}-SD_{f}}\right|$$ (3)

where *SD*_0_ is the *SD* value of soil sample at the RI state, *SD*_*f*_ is the *SD* value of sample at the FD state, and *SD* is the *SD* value of sample corresponding to a certain disturbance.

The purpose of this study is to evaluate the rationality and applicability of these two kinds of disturbance functions used in the research of soil damage mechanics by performing laboratory tests.

## Materials and methods

### Sample preparation

The expansive soils are collected from a channel slope in the location of South-to-North Water Transfer Project in Nanyang city, China (Longitude: 112.52, Latitude: 33.00). The soils were ground and sieved to 2 mm. Particles with the diameter less than 0.05 mm account for 85% of the total weight. The relative density is 2.71, liquid limit is 58.5%, and plastic limit is 22.8%. All specimens were initially compacted at the optimal water content of 24% and the dry density of 1.55 g/cm^3^. The diameter and height of the sample are 39.1 mm and 80 mm, respectively.

After the samples were repacked, the following artificial vertical cylindrical macro-pores were generated by pushing steel rods with the respective diameters into the samples: 1 macro-pore with diameter (∅) = 3 mm at the center of Sample B, 1 macro-pore with ∅ = 6 mm at the center of Sample C, 1 macro-pore with ∅ = 6 mm at the middle of the center and the edge (out-of-center) of Sample D, and 4 macro-pores with ∅ = 3 mm around the center of Sample E.

### CT-triaxial compression

The CT-triaxial testing equipment (S1 Fig.) used in this study includes the CT scanner and a computer-controlled triaxial test system. The CT machine is a spiral scanner type of Prospeed AL made by the GE Company. The technical details of the CT scanner are listed in Table 1. Consolidated drained triaxial compression tests at certain confining pressure (*σ*_3_ = 150*kPa*). The displacement rate of the axial loading punch was set at 0.0167 mm/min. The scanning was done four times during the entire test with the first one done after the completion of consolidation but before the shearing. The next three times of scanning were done with the axial strain = 5%, 10%, and 15%, respectively. Five sections, namely Sections a, b, c, d, and e shown in S2 Fig, were scanned from the bottom to top of the soil sample in each scanning.

**Table 1:** The technical parameters of the CT scanner.

Voltage / kv	Current / mA	Time / s	Thickness / mm	Image reconstruction matrix	Spatial resolution / mm
120	165	3	3	512×512	0.38

## Results and discussion

The quantifiable information, including *MD* value and *SD* value, was obtained using a software in conjunction with the CT equipment during different test stages. S3 Fig shows the macro-pore section under triaxial compression. It consists of five rows and four columns with each row corresponding to one sample. They represent Samples A, B, C, D, and E respectively from the top to bottom of the figure. Each column corresponds to one testing stage. The left to right lists the scanned samples by the CT equipment corresponding to the axial strains of 0%, 5%, 10%, and 15% respectively.

### Shear strength

The axial strain developed with the increasing deviator stress. The deviator stress is internal force divided by sectional area. Moreover, the deviator stress is effective stress that the bearing area reduction is taken into account since the samples have different type of macro-pores. The relationship between the deviator stress and the volumetric strain with the axial strain is shown in S4 Fig. As seen, the disturbed sample with macro-pore diameter of 3 mm has the highest shear strength and the sample without a macro-pore has the lowest value. Moreover, the difference in shear strength among the disturbed samples C, D, and E is small because the macro-pores in these samples have same size, despite of the different positions and qualities of macro-pores. This implies that the size rather than the position of the macro-pore primarily affects the shear strength of soil. The shear strength is higher for the sample with macro-pore disturbance than that without it. This is contrary to the general thinking that a disturbance would degrade the soil strength. The plausible explanation is that the soil becomes denser in the vicinity of macro-pores when the rod was inserted into the soil sample to create structural disturbance. It is similar to what found in the studies [5,6]. The volumetric strains of all samples are positive. Moreover, the volumetric strain of the sample with macro-pore disturbance is smaller than that found in the sample without macro-pore because the cylindrical macro-pores are contracted during compression. The macro-pore damage has been partly repaired during the compression.

Another set of tests are conducted to investigate the effect of macro-pores on the mechanical properties of soil samples with a different moisture content *w* of 7% which is lower than the plastic limit. It is found that the shear strength of macro-pore disturbed samples decreased compared to those with no macro-pore[12]. The macro-pore disturbance effect on soil strength strongly depends on the moisture content of soil. However, the main objective of this study is to investigate the relationship between disturbance effect on soil shear strength and macro-pore size.

### Quantitative morphometry

The *M* and *SD* values of the scanned CT images were also obtained through the use of the software accompanied with the CT equipment. The mean density and the discreteness of the soil sample at a scanned section are represented by the *M* and *SD* values, respectively. The variations of *SD* and *MD* values with axial strains are shown in S5 and S6 Figs.

As shown in S5 Fig, the standard deviation (i.e., *SD* value) decreases with an increasing axial strain, indicating that the discreteness of the scanned sample section decreases with increasing axial strains. This also implies that the soil is homogenized during the compression test. Moreover, the scale of *SD* variations is consistent among samples C, D, and E which have the same total macro-pore volume. In addition, the shear strength for these three samples is similar since they have the same macro-pore disturbance, as discussed above. Therefore, the *SD* value can be used as a key parameter to define the structural disturbance function of soil.

As shown in S6 Fig, the mean density (i.e., *MD* value) increases with increasing axial strains, meaning that the soil is densified during the compression test. However, the variations of *MD* values of the five samples with the axial strain are random, irrespective of different macro-pore volumes and positions. This indicates that the *MD* value is not suitable to characterize the magnitude of soil disturbance, nor to define the structural disturbance function of soil.

### Disturbance function

A further analysis on the relationship between structural disturbance functions (*D*_*1*_ and *D*_*2*_) and the magnitude of macro-pore disturbance is done in this study. As shown in S7 and S8 Figs, there is no definite correlation between the structural disturbance function *D*_*1*_ and the magnitude of macro-pore disturbance. However, the structural disturbance function *D*_*2*_ increases with the increasing magnitude of macro-pore disturbance. Moreover, *D*_*2*_ functions for the disturbed samples C, D, and E having the same macro-pore size display in a similar magnitude (difference < 19%). There seems to have a consistency between a value from the structural disturbance function *D*_*2*_ and the macro-pore volume, compared to the structural disturbance function *D*_*1*_. As a result, the structural disturbance function defined by the *SD* value is more reasonable and applicable than that defined by the *MD* value.

## Conclusions

Artificial cylindrical macro-pores disturbance with different macro-pore sizes and positions were generated in four compacted soil samples. The shear strength, the evolution of the *MD* and *SD* values subjected to shear deformation, the correlations between the structural disturbance functions *D*_*1*_ and *D*_*2*_, and the magnitude of macro-pore disturbance, were investigated using CT-triaxial tests under the same confining pressure and matric suction. The size rather than the position of macro-pore disturbance primarily affects the shear strength of repacked soil. The macro-pore disturbance effect on soil strength depends on the size of macro-pore and the moisture content of soil. The coupling effect between the moisture content and the disturbance degree on the strength of soil is a far more complicated issue deserving for a further study.

The structural disturbance function defined using the *SD* value is more reasonable and applicable to study the soil strength at a disturbed state. On the other hand, the *MD* value is not suggested to characterize the magnitude of soil disturbance.

## Supporting information

S1 Fig The CT-triaxial test equipment used in the current study. (TIF)

S2 Fig The position arrangement of scanned sections. (TIF)

S3 Fig The CT scanned images for Samples A to E taken at Section c during the triaxial shearing test (Left to right: axial strain = 0%, 5%, 10%, and 15%, respectively; Top to bottom: Samples A, B, C, D, and E, respectively).
(TIF)

S4 Fig The relationship between deviator stress *q*, volumetic strain *ε*_*v*_ and axial strain *ε*_*a*_ (i.e., *q* = *σ*_1_−*σ*_3_ with *σ*_1_ = *the axial stress* and *σ*_3_ = *the radial stress*, respectively; the radial stress *σ*_3_ = *confining pressure* also). (TIF)

S5 Fig Variations in SD values with axial strains. (TIF)

S6 Fig Variations in MD values with axial strains. (TIF)

S7 Fig Variation in structural disturbance function D_1_ with different magnitudes of macro-pore disturbance. (TIF)

S8 Fig Variation in structural disturbance function D_2_ with different magnitudes of macro-pore disturbance. (TIF)

S1 Dataset [Data for S4 Fig]. (OPJ)

S2 Dataset [Data for S5 Fig and S6 Fig]. (OPJ)

S3 Dataset [Data for S7 Fig and S8 Fig]. (OPJ)
