# Supplementary figures and images for: Disturbance function for soil disturbed state strength based on X-ray computed tomography triaxial test
Source: PLoS One. 2019 May 2;14(5):e0215961. doi: 10.1371/journal.pone.0215961 (PMC6497377; doi:10.1371/journal.pone.0215961)

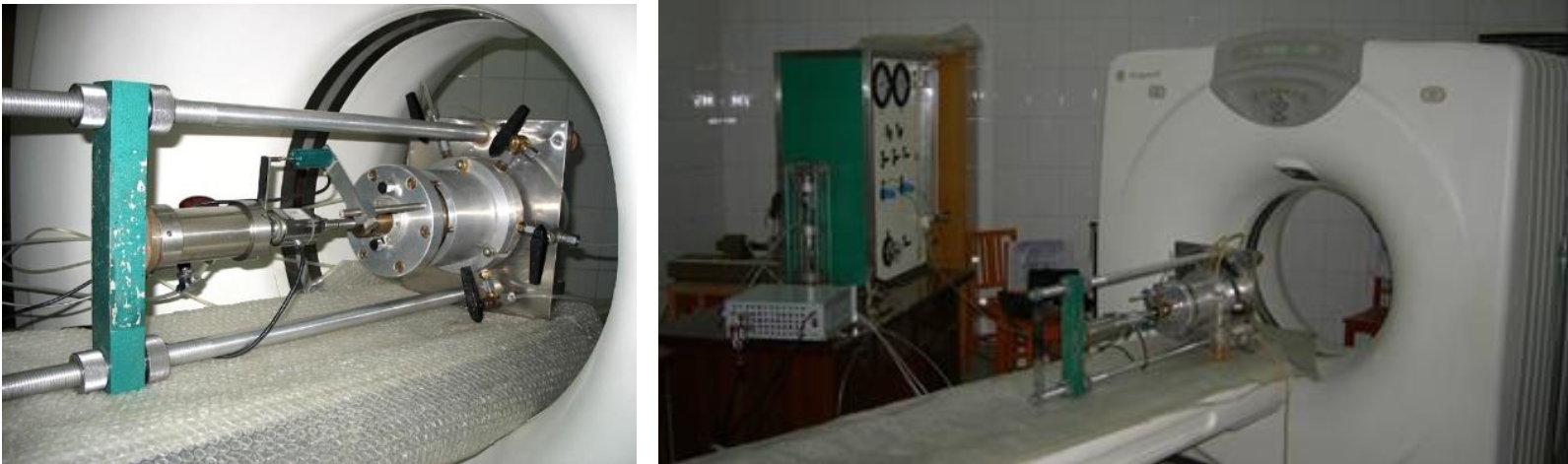

Supplement: S1 Fig — (TIF) [file pone.0215961.s001.tif]

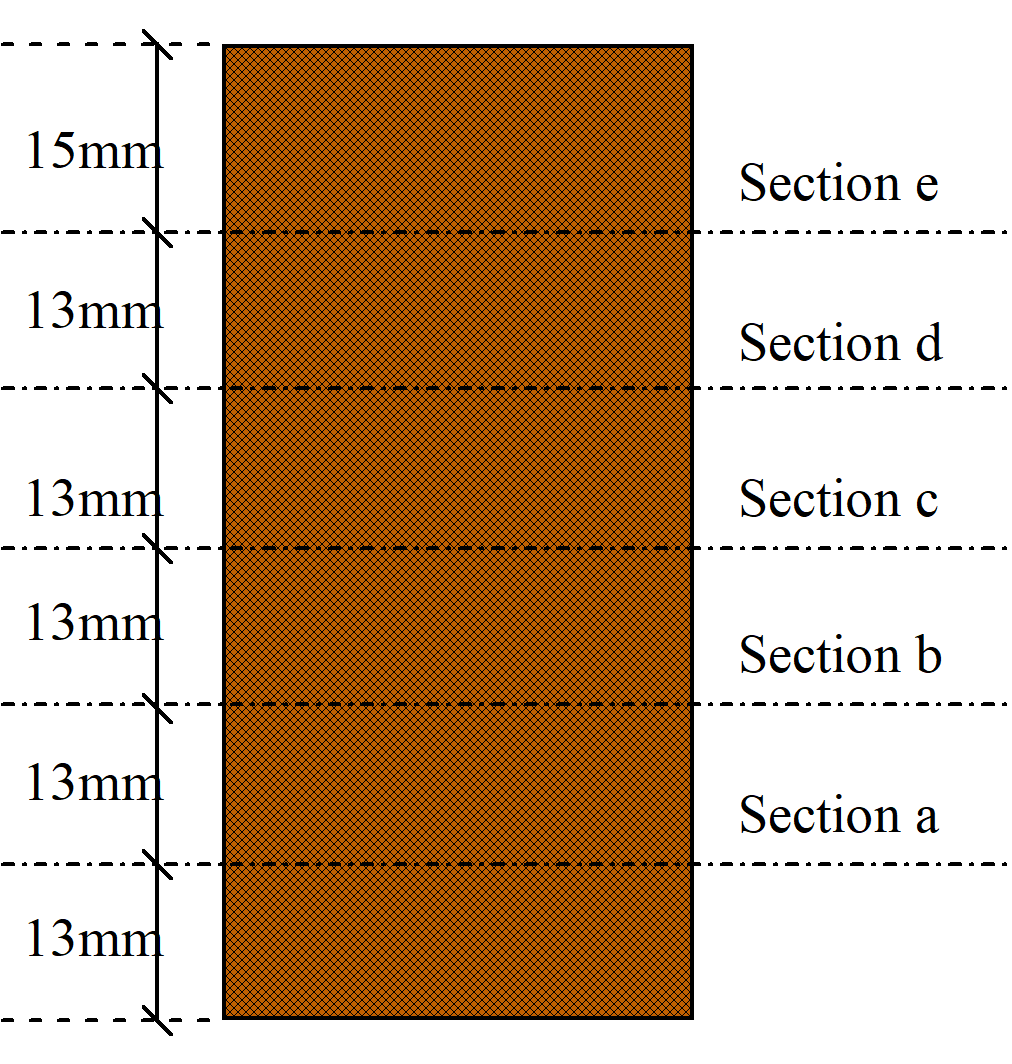

Supplement: S2 Fig — (TIF) [file pone.0215961.s002.tif]

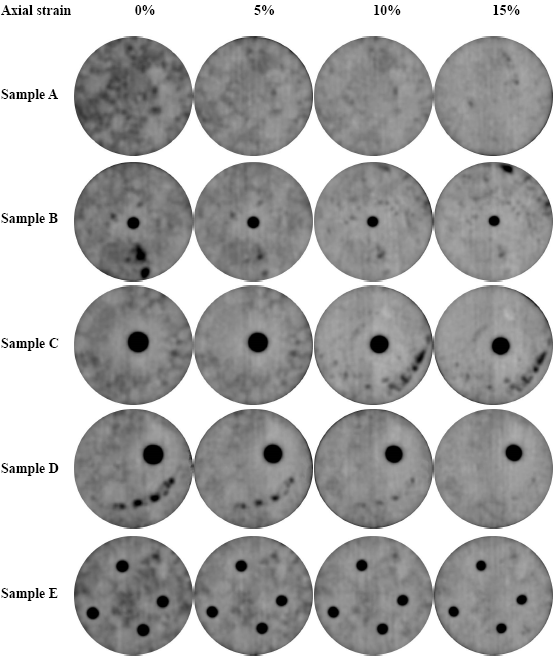

Supplement: S3 Fig — The CT scanned images for Samples A to E taken at Section c during the triaxial shearing test (Left to right: axial strain = 0%, 5%, 10%, and 15%, respectively; Top to bottom: Samples A, B, C, D, and E, respectively). (TIF) [file pone.0215961.s003.tif]

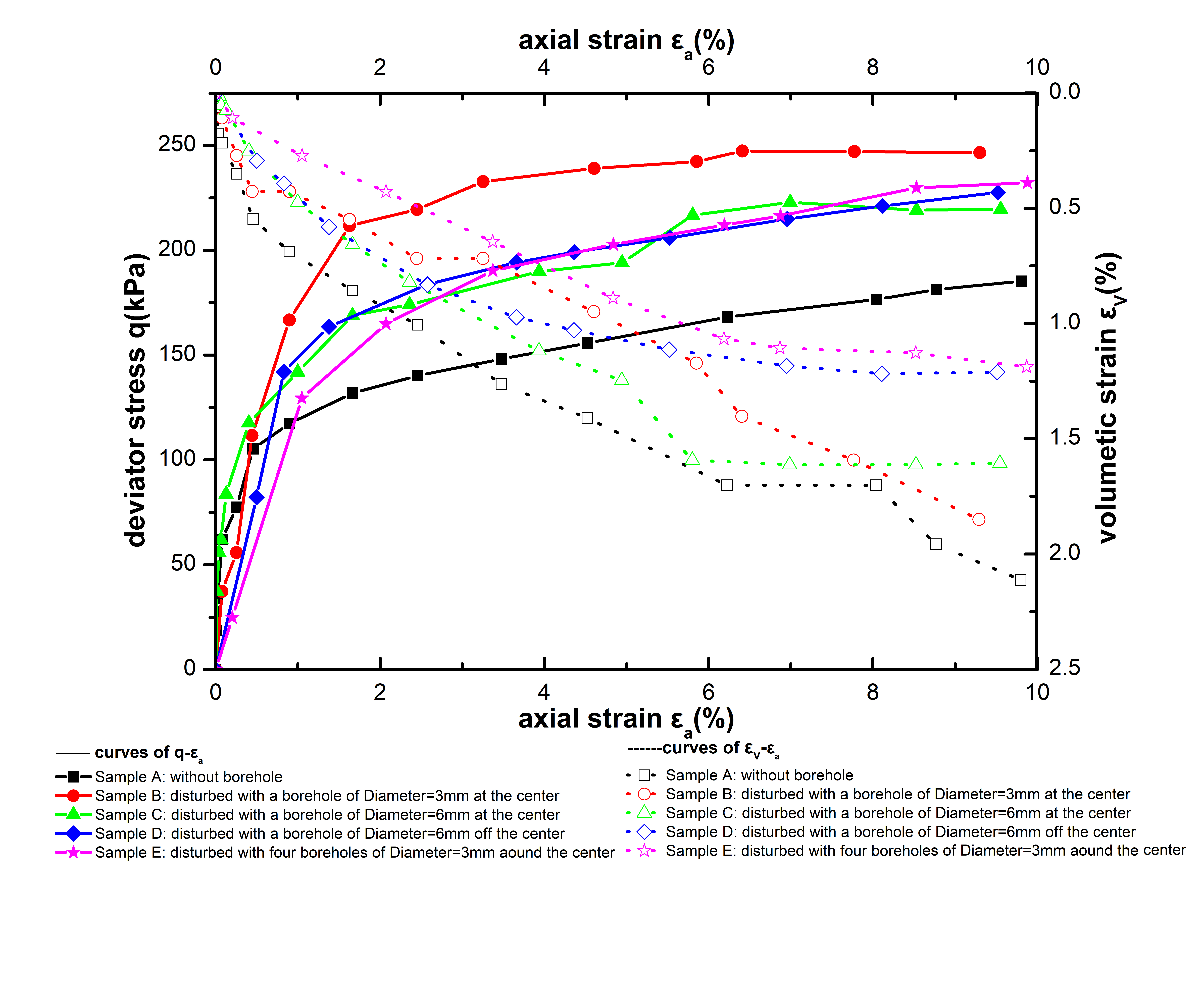

Supplement: S4 Fig — (TIF) [file pone.0215961.s004.tif]
